# The Rationale for Delaying Aging and the Prevention of Age-Related Diseases

**DOI:** 10.5041/RMMJ.10087

**Published:** 2012-10-31

**Authors:** Nir Barzilai, Gad Rennert

**Affiliations:** 1 The Ingeborg and Ira Leon Rennert Chair of Aging Research, Professor of Medicine and Molecular Genetics; Director, Institute for Aging Research, Albert Einstein College of Medicine, Bronx, NY, USA;; 2 Department of Community Medicine and Epidemiology, Carmel Medical Center and B. Rappaport Faculty of Medicine, Technion-Israel Institute of Technology, Haifa, Israel; and; 3 Office of the Chief Physician, Clalit Health Services, Tel Aviv, Israel

## Age as a risk for diseases:

**Figure 1:**
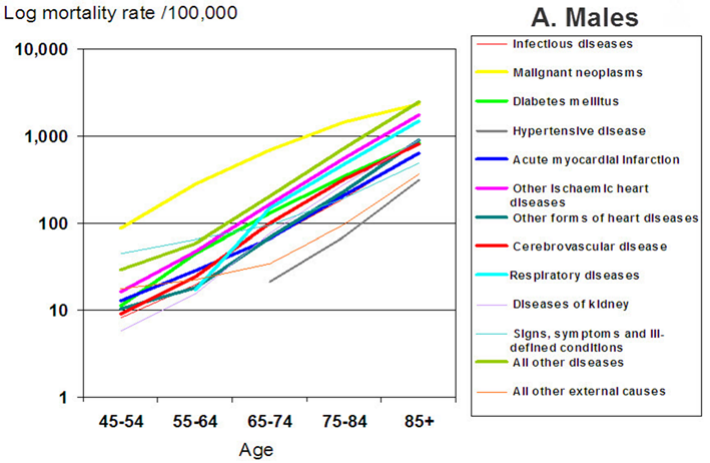

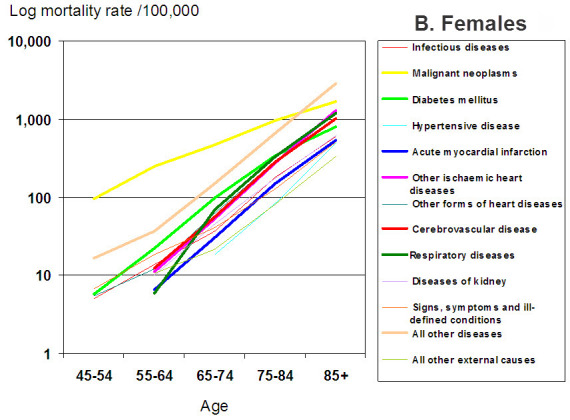
**Mortality rates for major causes of death, by age, and gender (A: Males; B: Females), Israel 2009.**

We offer a different approach to delaying or preventing age-related diseases. To understand the necessity for a new approach we have plotted the mortality rates in Israelis in relation to specific age groups and diseases. With the common phenomenon of aging of Western populations it is of utmost importance to follow time-dependent and age-dependent mortality patterns to predict future needs of Western health systems. Age-specific, gender-specific, and cause-of-death-specific mortality rates were extracted from the statistical abstract of Israel^1^ and include data for the period of 1975–2010; these are presented in Figure 1, separately for men (A) and women (B). Detailed age-specific causes of death data were available for the year 2009. Data presented were restricted to 5-year age groups starting at age 50, and for cause-specific mortality to the following age groups: 45–54, 55–64, 65–74, 75–84, and 85+. Causes of mortality were separated into malignant diseases, acute myocardial infarction, other ischemic heart diseases, other forms of heart diseases, cerebrovascular disease, diabetes mellitus, respiratory diseases, diseases of kidney, infectious diseases, all external causes, signs/symptoms and ill-defined conditions, and all other diseases. Figure 1 is similar to the one posted on the National Institute of Aging website and similar to data across the industrial world. The striking feature of this graph is that aging is a major log scale risk for most diseases, including the major killers: heart disease, cancer, diabetes, and Alzheimer’s. For example, while aging is a 100-fold risk for cardiovascular disease (CVD) according to Figure 1, hypercholesterolemia is known to carry only a three-fold risk for CVD. For each of the mentioned diseases, aging is a log risk greater than the most important known risk factor for that disease.

## What is the interpretation of this relationship of age and diseases?

Based on Figure 1, those of us who investigate the biology of aging have hypothesized that unless we delay aging, we will not have a major impact on age-related diseases. Even if all cardiovascular disease were to be eliminated, the expected impact would be an additional 2.87 years of life.^2^ Explaining this in part is that cardiovascular disease can be prevented by drugs, and patients have been saved by interventions such as coronary vessel stenting and by-pass surgery. However, those “saved” patients are likely to die from diabetes, cancer, or Alzheimer’s disease (if not from a second cardiovascular event) within a couple of years.^3^ This is because we have not addressed the aging part, which continues to put us at risk for other age-related diseases. Unless we delay aging, we will mainly replace one disease with another. Thus, addressing aging overall and not just aiming to prevent a single disease, may lead to a longer health span, and may be more economically cost-effective as well.

## What then is the evidence suggesting that advances in scientific discoveries make this discussion relevant at this time?

First, several interventions such as caloric restriction and modulating the growth hormones pathway have resulted in extending life-span of mammalian models, suggesting that the study of the mechanism of action in these interventions may be relevant for human aging.^4^ Second, an intuitive question to ask is: Do we humans age at different rates? This is a question I have asked thousands of laypeople and scientists during lectures including during the Barzilai Symposium on Aging held at Rambam Health Care Campus in March 2011. Nearly 100% of all audiences have said yes. Intuitively, we recognize that some 50-year-old people look like they are 40 and some look like they are 60. This variability serves as a unique opportunity for us to understand the biology of aging and try to modulate it. Following up on the notion that humans age at different rates, a novel approach has looked for genetic factors that allow animals, centenarians, and other elderly with good health to live longer. Several single gene manipulations have led to longevity in lower species, and some of these genes have been implicated in human longevity.^5^ In addition, several human gene variants have been associated with exceptional longevity whether by a candidate genes or unbiased approaches.^6^–^8^

Most important, several drug therapies that have been suggested to prolong healthy aging and even life-span in animals are being used experimentally in humans.^9^ These include rapamycin (mTOR inhibitor), sirtuins (such as resveratrol), humanin (mitochondrial peptide), and cholesterylester transfer protein inhibitors (which increase the good HDL-cholesterol). These candidate agents are all undergoing drug development, are in specific clinical trials, or phase 3 trials by large pharmaceutical companies, suggesting that drugs affecting aging may be available for us soon.

In summary, a cost-effective way to prevent many diseases is to delay the aging process. Such an approach is necessary, feasible, and already has examples of success.

## Abbreviations:

CVD: cardiovascular disease.

## References

[1] (2011). Statistical Abstract of Israel 2011. Central Bureau of Statistics.

[2] Beltrán-Sánchez H, Preston SH, Canudas-Romo V (2008). An integrated approach to cause-of-death analysis: cause-deleted life tables and decompositions of life expectancy. Demogr Res.

[3] Eriksson UK, Bennet AM, Gatz M, Dickman PW, Pedersen NL (2010). Nonstroke cardiovascular disease and risk of Alzheimer disease and dementia. Alzheimer Dis Assoc Disord.

[4] Barzilai N, Bartke A (2009). Biological approaches to mechanistically understand the healthy life span extension achieved by calorie restriction and modulation of hormones. J Gerontol A Biol Sci Med Sci.

[5] Suh Y, Atzmon G, Cho MO (2008). Functionally-significant insulin-like growth factor-I receptor mutations in centenarians. Proc Natl Acad Sci U S A.

[6] Barzilai N, Atzmon G, Schechter C (2003). Unique lipoprotein phenotype and genotype associated with exceptional longevity. JAMA.

[7] Atzmon G, Rincon M, Schechter CB (2006). Lipoprotein genotype and conserved pathway for exceptional longevity in humans. PLoS Biol.

[8] Sebastiani P, Solovieff N, Dewan AT (2012). Genetic signatures of exceptional longevity in humans. PLoS One.

[9] Barzilai N, Huffman DM, Muzumdar RH, Bartke A (2012). The critical role of metabolic pathways in aging. Diabetes.

